# The need for a cancer exposome atlas: a scoping review

**DOI:** 10.1093/jncics/pkae122

**Published:** 2024-12-19

**Authors:** Anna S Young, Catherine E Mullins, Neha Sehgal, Roel C H Vermeulen, P Martijn Kolijn, Jelle Vlaanderen, Mohammad L Rahman, Brenda M Birmann, Dinesh Barupal, Qing Lan, Nathaniel Rothman, Douglas I Walker

**Affiliations:** Gangarosa Department of Environmental Health, Rollins School of Public Health, Emory University, Atlanta, GA 30322, United States; Gangarosa Department of Environmental Health, Rollins School of Public Health, Emory University, Atlanta, GA 30322, United States; Gangarosa Department of Environmental Health, Rollins School of Public Health, Emory University, Atlanta, GA 30322, United States; Institute for Risk Assessment Sciences, Utrecht University, Utrecht 3584 CM, The Netherlands; Institute for Risk Assessment Sciences, Utrecht University, Utrecht 3584 CM, The Netherlands; Julius Global Health, The Julius Center for Health Sciences and Primary Care, University Medical Center, Utrecht 3584 CG, The Netherlands; Institute for Risk Assessment Sciences, Utrecht University, Utrecht 3584 CM, The Netherlands; National Cancer Institute, Bethesda, MD 20892, United States; Channing Division of Network Medicine, Department of Medicine, Brigham and Women’s Hospital and Harvard Medical School, Boston, MA 02115, United States; Department of Environmental Medicine and Climate Science, Icahn School of Medicine at Mount Sinai, New York, NY 10029, United States; National Cancer Institute, Bethesda, MD 20892, United States; National Cancer Institute, Bethesda, MD 20892, United States; Gangarosa Department of Environmental Health, Rollins School of Public Health, Emory University, Atlanta, GA 30322, United States

## Abstract

**Background:**

Despite advances in understanding genetic susceptibility to cancer, much of cancer heritability remains unidentified. At the same time, the makeup of industrial chemicals in our environment only grows more complex. This gap in knowledge on cancer risk has prompted calls to expand cancer research to the comprehensive, discovery-based study of nongenetic environmental influences, conceptualized as the “exposome.”

**Methods:**

Our scoping review aimed to describe the exposome and its application to cancer epidemiology and to study design limitations, challenges in analytical methods, and major unmet opportunities in advanced exposome profiling methods that allow the quantification of complex chemical exposure profiles in biological matrices. To evaluate progress on incorporating measurements of the exposome into cancer research, we performed a review of such “cancer exposome” studies published through August 2023.

**Results:**

We found that only 1 study leveraged untargeted chemical profiling of the exposome as a method to measure tens of thousands of environmental chemicals and identify prospective associations with future cancer risk. The other 13 studies used hypothesis-driven exposome approaches that targeted a set of preselected lifestyle, occupational, air quality, social determinant, or other external risk factors. Many of the included studies could only leverage sample sizes with less than 400 cancer cases (67% of nonecologic studies) and exposures experienced after diagnosis (29% of studies). Six cancer types were covered, most commonly blood (43%), lung (21%), or breast (14%) cancer.

**Conclusion:**

The exposome is underutilized in cancer research, despite its potential to unravel complex relationships between environmental exposures and cancer and to inform primary prevention.

## Graphical Abstract

**Graphical Abstract:**
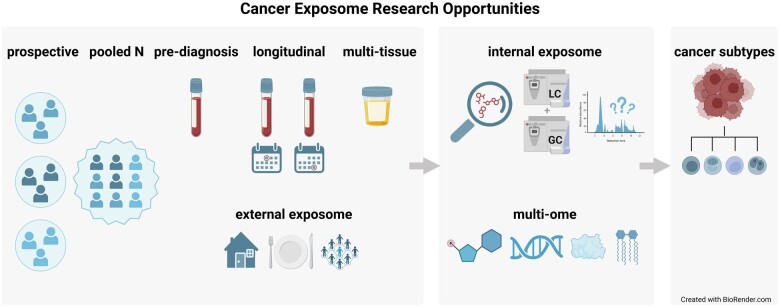

## Introduction

### Environmental origins of cancer

Cancer has historically been studied as a genetic disease.^1^ Tremendous advances have been realized in the genomic characterization of cancer, but genetic risk factors are mostly limited to highly penetrant disease variants and to polygenic risk scores summarizing tens to hundreds of gene mutations.^2^^,^^3^ We now know that the average heritability of cancer is only about 10%, with nongenetic factors, including environmental exposures, likely initiating the other 90% of cancer cases.^4^ Gene–environment interactions may drive a substantial proportion of this missing heritability of cancer, but the environmental component remains largely unknown and unmeasured.^5-7^ Environmental exposures can be important drivers of risk during all phases of cancer development (initiation, promotion, and progression) and through a diverse array of genotoxic or nongenotoxic modes of action.^8^^,^^9^ The development of advanced approaches now allowing systematic characterization of environment and its role in cancer offers important opportunities for the primary prevention of cancer.

### Complexity of environmental exposures

Over a lifetime, humans are exposed to a complex and heterogenous mixture of chemical exposures, including environmental contaminants, commercial products, dietary chemicals, and other classes of compounds. The International Agency for Research on Cancer (IARC) has classified 546 agents as known, probable, or possible human carcinogens,^10^ but at least 355 000 chemicals or mixtures have been registered for production and use around the world, including about 69 000 chemicals in the past decade alone.^11^ Many of these have limited, if any, toxicological assessment, and understanding of how they interact within mixtures is even less understood. Roughly 15% of these registered chemicals are not identifiable because of confidential business information, challenging prediction of persistence and toxicological effects.^11^ When considering additional isomers, impurities, and potential biotransformation products arising from these chemicals, the full potential for human exposures likely exceeds 1 million, many of which are unmeasured, uncharacterized, and unknown.^12^

**Figure 1.**
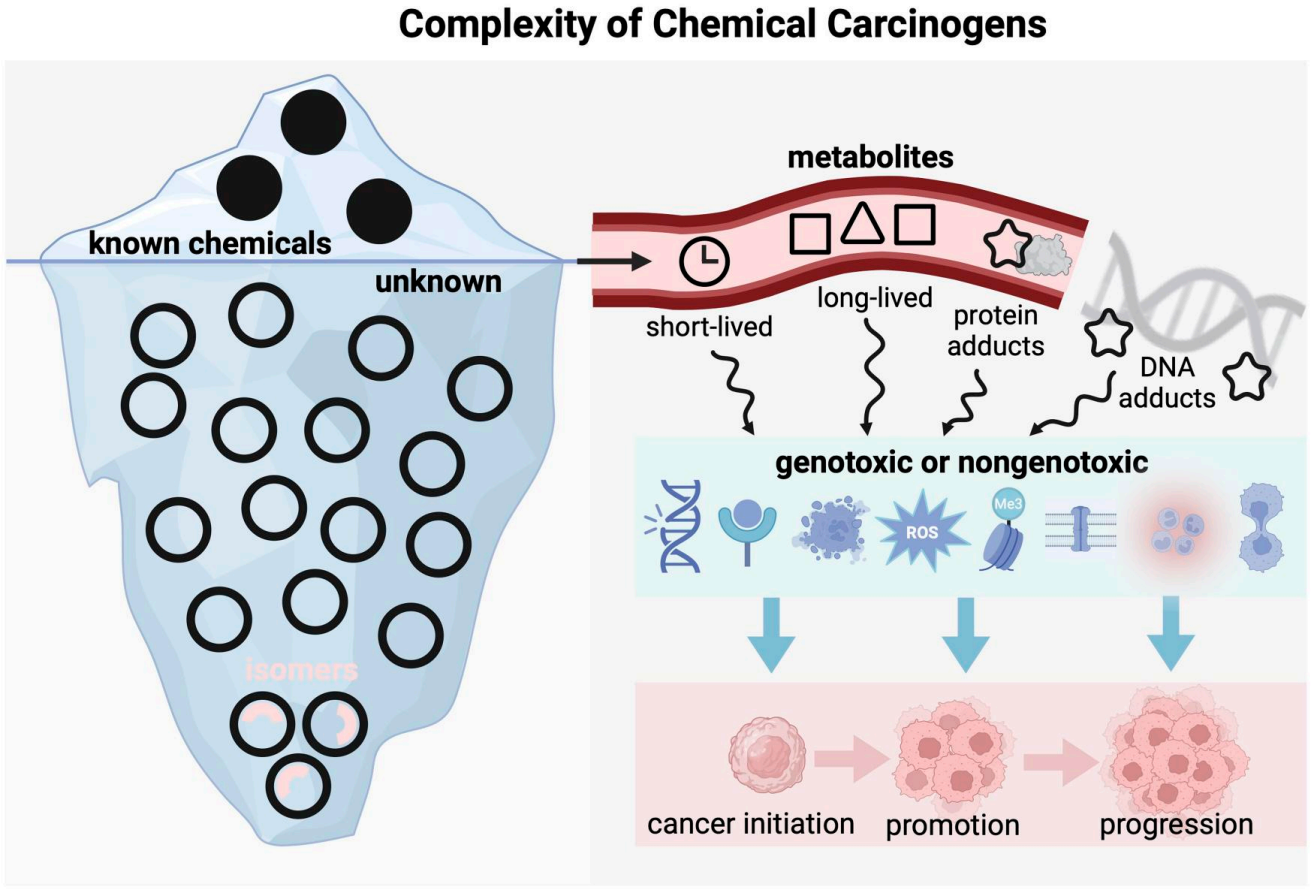
Diagram of select characteristics and mechanisms of environmental chemical carcinogens demonstrating their complexity for research study design. Note: This is not comprehensive of all physiological mechanisms.

Risk assessment of chemical carcinogenicity has been challenged by this large number of chemicals and their transformation products, diverse mechanisms of carcinogenicity, long latencies in cancer development, and limited analytical capacity (Figure 1). For example, traditional targeted chemical methods have considered only a limited number of chemical exposures in large human biomonitoring programs.^13^ Although more interpretable for regulatory purposes, targeted approaches can measure only a small number of exposure biomarkers identified a priori, and scaling up to higher numbers of exposures is limited by cost, time, and availability of validated protocols.^14^ In addition, nongenotoxic carcinogens that do not directly damage or interact with DNA are left unidentified in traditional carcinogenicity assessments based on genotoxicity or mutagenicity outcomes.^9^ Even among chemicals classified by IARC as known human carcinogens, a review in 2009 found that 17% (of 77) tested negative in genotoxicity assays and 27% negative in mutagenicity assays.^15^ The variety of nongenotoxic mechanisms include epigenetic alterations, oxidative stress, chronic inflammation, immune suppression, disruption of receptors or their ligands, and cell immortalization, and their importance and complexity are increasingly being recognized in the investigation of environmental drivers of cancer.^16^

### Defining the exposome

Despite the importance of considering environment in the missing heritability of cancer, there is an imbalance in the degree of methodological development applied to the characterization of environmental exposures relative to genomics.^17^ This gap in cancer research prompted Christopher Wild in 2005 to first introduce the exposome, defined as a complement to the genome.^17^ The exposome concept underscores the importance of an individual’s lifespan history of environmental exposures in defining future disease risk, which, unlike the genome, are not fixed and can vary at multiple scales. A more practical definition was recently proposed to be the study of “the comprehensive and cumulative effects of physical, chemical, biological, and psychosocial influences that impact biological systems by integrating data from a variety of interdisciplinary methodologies and streams to enable discovery-based analysis of environmental influences on health.” This refinement advocates for systematic, discovery-based approaches that can generate new hypotheses about previously unidentified environmental risk factors rather than focusing solely on a small number of known exposures.^18^

Untargeted high-resolution mass spectrometry (HRMS) is a critical advancement in discovery-based exposome methodologies that supports the systematic, omic-scale measurement in biospecimens of both the chemical exposome (environmentally derived chemicals and their biotransformation products) and the metabolome (endogenous biological response profiles) in a single analytical run.^19-22^ This cumulative “internal exposome” focuses on in vivo measurements, as an external environmental stressor that alters an individual’s disease risk would have interacted with an internal target and resulted in a biological change.^20^ HRMS now allows detection of upwards of 100 000 signals in biological specimens, including exogenous environmental chemicals, endogenous metabolites, microbiome-related compounds, and pharmaceuticals.^23^ For optimal detection of exogenous chemicals across a wide spectrum of physical–chemical properties, both liquid chromatography (LC) and gas chromatography (GC) HRMS can be used.^13^^,^^24^ These untargeted methods have become more accurate, inexpensive, higher-throughput, feasible for smaller sample volumes, and more streamlined through data pipelines.^25-28^ In the data output, the mass spectral features of detected signals are elucidated and can be evaluated for disease associations, whether or not their chemical identities are known.^6^ Untargeted analysis offers the opportunity to characterize exposures to previously unidentified chemicals or their metabolites and to capture biomarkers of exposures that may have occurred years earlier.^29^

Despite constantly evolving technology to investigate the exposome, there is a lack of synthesized information on how it has been applied to the study of cancer. The objectives of our scoping review were to evaluate available literature on the human cancer exposome, to characterize commonalities in study design and exposome assessment methods, and to identify current challenges and unmet research opportunities.

## Methods

### Review protocol

Our scoping review was written in accordance with the Preferred Reporting Items for Systematic Reviews and Meta-Analysis Protocols—Extension for Scoping Reviews (PRISMA–ScR).^30^ We registered the protocol with Open Science Framework on August 9, 2023 (doi.org/10.17605/OSF.IO/4PYUB).

### Eligibility criteria

Our search on August 10, 2023, used 3 databases that we filtered to English: PubMed/MEDLINE, Embase, and Web of Science: Core Collection. We included published (or preprint) scientific journal articles of original research evaluating the exposome in relation to outcomes of cancer in human participants. To evaluate study eligibility in terms of the exposome, we focused on studies assessing *environmental exposures* using any of the following methods: (1) specifically described as exposomic in the title or abstract (regardless of the definition or use of biospecimens), (2) untargeted chemical analysis, (3) suspect screening chemical analysis, (4) untargeted chemical adductomics, or (5) untargeted metabolomics with discussion of identified chemicals in abstract, for which we defined *chemicals* as exogenous xenobiotics. Adductomics refers to the measurement of adducts formed when reactive electrophiles generated from the metabolism of chemicals bind to blood proteins or DNA.^31^^,^^32^ During full-text review, we removed metabolomics studies not addressing or intending to assess environmental exposure with their methods, even if in the results or discussion the authors identified environmental chemicals that they happened to detect in samples.

### Study selection and data extraction

Using Covidence software (Veritas Health Innovation), each study’s title and abstract were screened by 2 reviewers. Any disagreements in study inclusion were independently resolved by a third reviewer, with discussion and consensus as needed. The same process was then conducted for the full-text screening phase. We grouped the included studies by their exposome approach, exposure type, exposure timing, cancer outcome measure, and study design. All visualizations were generated using R (version 4.3.1). Additional details on our methods are provided in the Supplementary Material.

## Results

### Characteristics of selected studies

**Figure 2.**
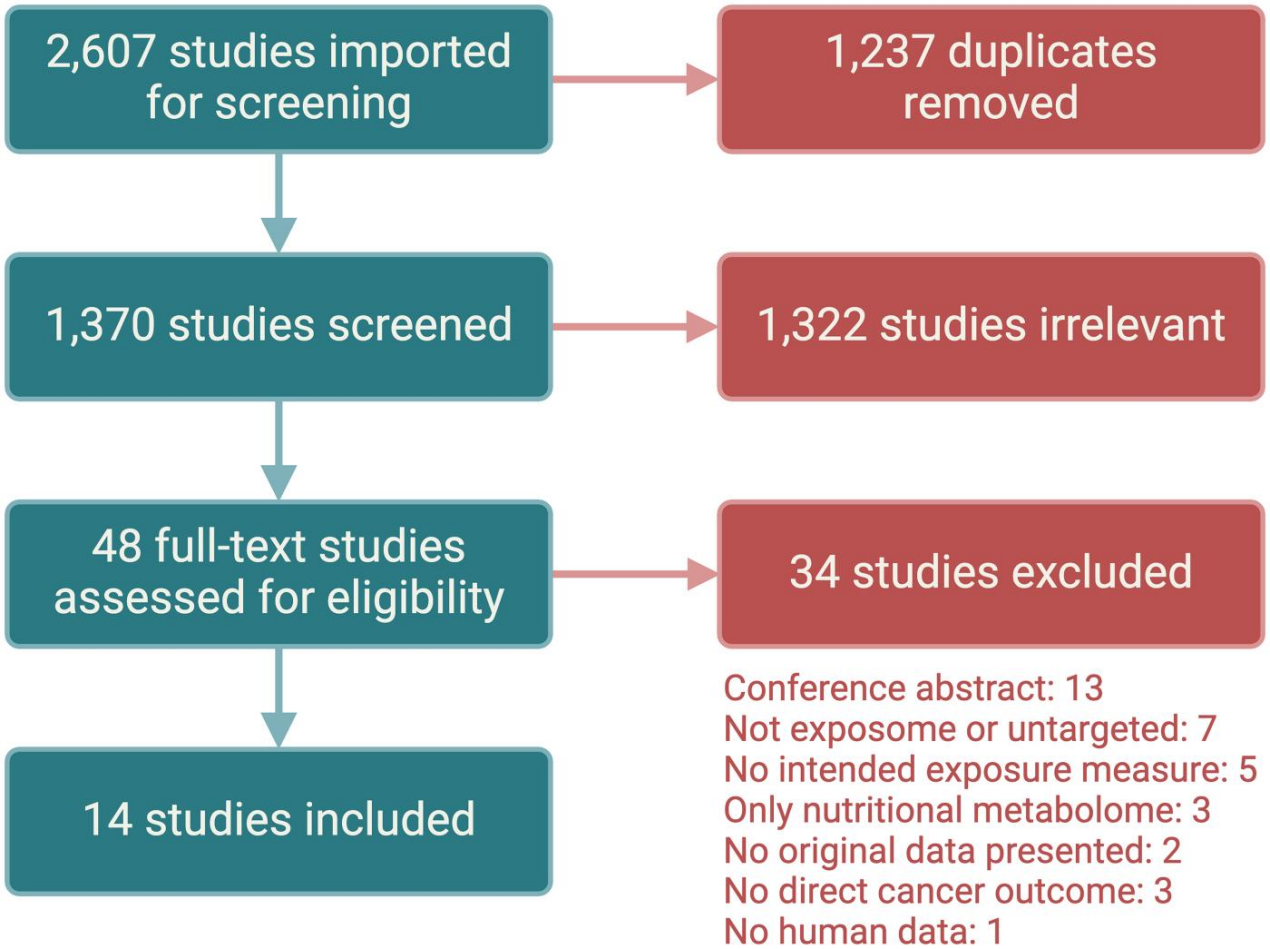
Flow chart of number of studies screened, excluded, and assessed in our scoping review.

In total, 1370 studies were initially screened, 48 full-text articles were assessed for eligibility, and 14 were deemed eligible (Figure 2). Table 1 summarizes characteristics of the 14 studies.^33-46^

**Table 1.** Summary of 14 reviewed studies and their methods related to the cancer exposome.

	Study design				Outcome		
Study	Relevant aim(s)	Study design	N	No. of cases	Endpoint	Cancer	Subtypes
Belloni 2020	Statistical mixtures method development	Cohort	3377	94	Mortality	Lung	No
Boyle 2022	Statistical mixtures method development; exposures associated with cancer risk	Case–control^a^	2378	1321	Diagnosis	Lymphoma	No
Chen 2022	Exposures associated with cancer risk	Cohort	335 370	10 702	Diagnosis	Colorectal	No
Faisandier 2011	Statistical mixtures method development; exposures associated with cancer risk	Case-only	77	77	Diagnosis	Lymphoma	No
Go 2023	Exposure biomarkers and metabolic biomarkers associated with cancer risk	Nested case–control^b^	566	182	Diagnosis	Breast	No
Guo 2022	Exposure biomarkers associated with cancer severity	Case-only	12	12	Tissue state	Prostate	No
Hosnijeh 2021	Exposures associated with cancer risk	Cohort	475 426	2402	Diagnosis	Lymphoma	Yes
Juarez 2017	Exposures associated with cancer mortality; disparities in cancer mortality	Ecologic	2067	N/A	Mortality	Lung	No
Rahman 2023	Exposure biomarkers associated with cancer risk	Nested case–control^c^	155	52	Diagnosis	Leukemia	No
Stevens 2023	Exposures and metabolic biomarkers associated with cancer risk	Case–cohort	3678	1709	Diagnosis	Breast	No
Wheeler 2021	Statistical mixtures method assessment; exposures associated with cancer risk	Case–control^c^	564	268	Diagnosis	Leukemia	No
Yano 2020	Exposure biomarkers associated with cancer risk	Case–control^c^	782	386	Diagnosis	Leukemia	Yes
Zaccari 2022	Exposures associated with cancer risk and cancer severity	Case–control^c^	669	223	Diagnosis	Ampullary	Yes
Zhu 2023	Exposures associated with cancer risk	Ecologic	212	N/A	Diagnosis	Lung	No

Exposome								Population		
Main exposure topics	Approach	Hypotheses	No. of exposures	Platform	Specimens	Repeats	Exposure lag	Ages	Countries	Race or ethnicity info
Occupational; radiation	Exposome	A priori	3	None	None	No	Before outcome	Adults	France	Not reported
Chemicals; spatial	Exposome	A priori	27	GC-MS	House dust	No	After outcome	Adults	United States	Limited^d^
Lifestyle; diet; social; built/natural	Exposome	A priori	12	None	None	No	Before outcome	Adults	United Kingdom	Not reported
Occupational	Exposome	A priori	86	None	None	No	At time of outcome	Adults	France	Not reported
Chemicals	Exposome	Discovery	Untargeted	LC-MS	Serum	Yes	Before outcome	Adults	United States	Reported
Chemical adducts; diet	DNA adductome	Discovery	Untargeted	LC-MS	Tissue	No	After outcome	Adults	United States	Reported
Lifestyle; diet; social; medical	Exposome	A priori	84	None	None	No	Before outcome	Adults	Denmark, France, Germany, Greece, Holland, Italy, Norway, United Kingdom, Spain, Sweden	Not reported
Social determinants; lifestyle; occupational; built/natural; air pollution; chemicals; medical	Exposome	Hybrid	2162	None	None	No	Before outcome	Mixed	United States	Reported
Chemical adducts	Protein adductome (HSA)	Discovery	Untargeted	LC-MS	Serum/plasma	No	Before outcome	Adults	United States	Reported
Metabolites	Metabolome	Discovery	Untargeted	LC-MS	Plasma	No	Before outcome	Adults	United States	Reported
Chemicals	Exposome	A priori	49	GC-MS	House dust	No	After outcome	Children	United States	Limited^d^
Chemical adducts	Protein adductome (HSA)	Discovery	Untargeted	LC-MS	Newborn dried blood spots	No	Before outcome	Children	United States	Reported
Lifestyle; medical	Exposome	A priori	20	None	None	No	At time of outcome; before outcome	Adults	Italy	Not reported
Air pollution	Exposome	A priori	109	None	None	No	Before outcome	Mixed	United States	Reported

Abbreviations: GC = gas chromatography; HSA = human serum albumin (1 type of protein commonly analyzed in adductomics); LC = liquid chromatography; MS = mass spectrometry; N = final sample size used in analysis of the part of the study eligible for our scoping review; platform = laboratory analytical instrumentation. References.^33-46^

a Controls selected on the basis of frequency matching to cases.

b Controls selected randomly among non-cases.

c Controls selected on the basis of individual matching to cases.

d Two statistical method studies were defined as having “limited” race/ethnicity information because they used the data (eg, as a covariate) but did not report the population proportions.

### Synthesis of results

#### Cancer outcomes

**Figure 3.**
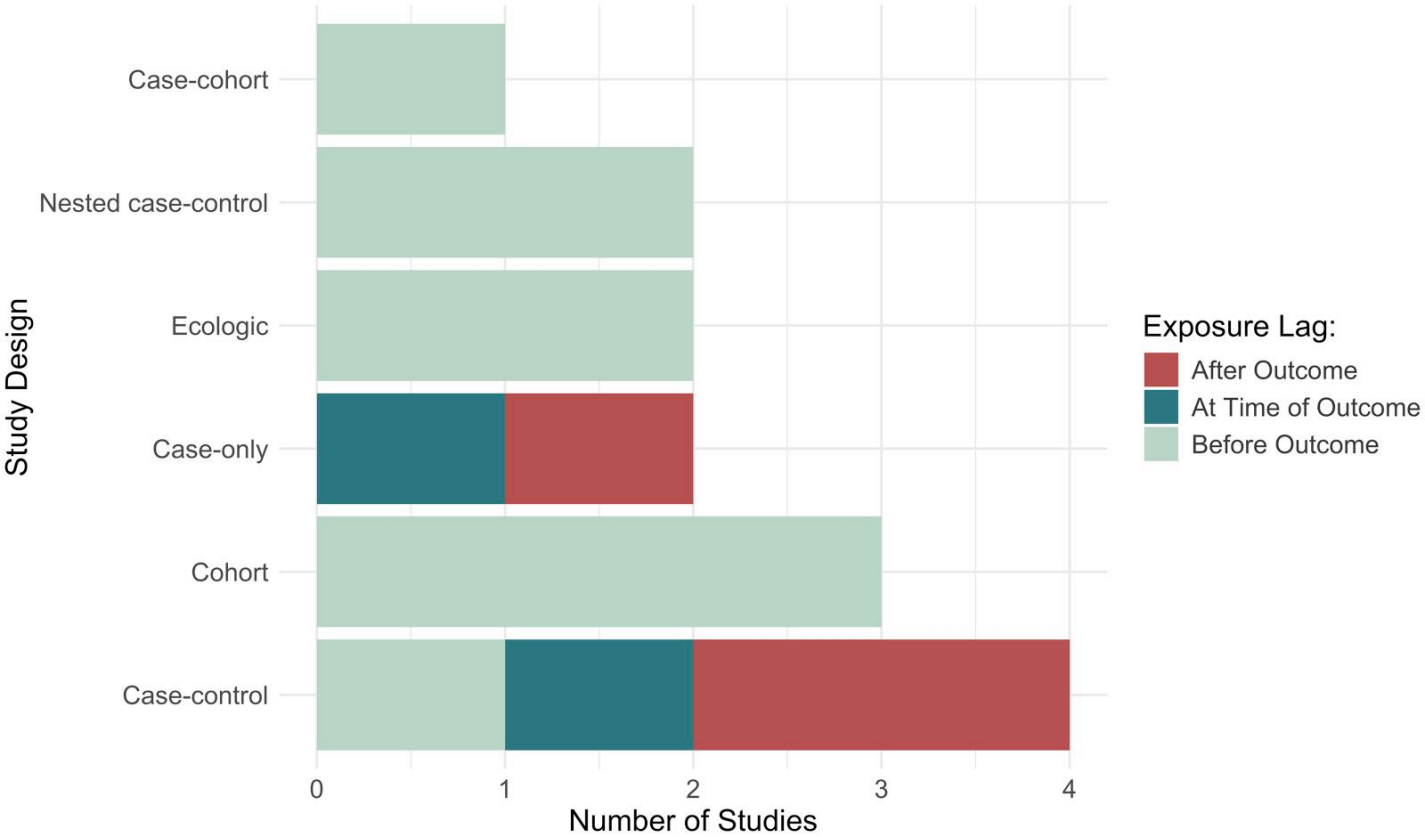
Case counts, cancer type, exposure types, and exposure methodological approach for the 14 cancer exposome studies analyzed in this scoping review. Points are labeled with the cancer type: Br = breast, Pr = prostate, Lu = lung, Le = leukemia, Ly = lymphoma, Co = colorectal. Notes: For ecologic study designs in two papers, the number of cases was treated as the number of sampled regions. For exposure type, the primary focus was chosen.

The 14 studies covered 6 cancer types (Table 1; Figure 3). The most common types were blood cancers (43%: 6 studies, including 3 of lymphoma and 3 of leukemia), lung cancer (21%: 3 studies), and breast cancer (14%: 2 studies). Only 1 study each investigated prostate, ampullary, and colorectal cancer. The cancer outcome was usually cancer diagnosis (79%: 11 studies), whereas another 2 studies (14%) evaluated cancer mortality and 1 study (7%) differentiated tissue states (low vs high cancer grades). Only 3 studies (21%) statistically analyzed individual subtypes of the cancer (Table 1).

#### Study design

The majority of eligible articles studied populations of mostly adult age (86%: 12) and within the United States (64%: 9) or Europe/United Kingdom (36%: 5) (Table 1). The availability of race/ethnicity information was inconsistent: half of the studies (50%: 7) reported or re-reported demographic data on race or ethnicity in the main article.

**Figure 4.**
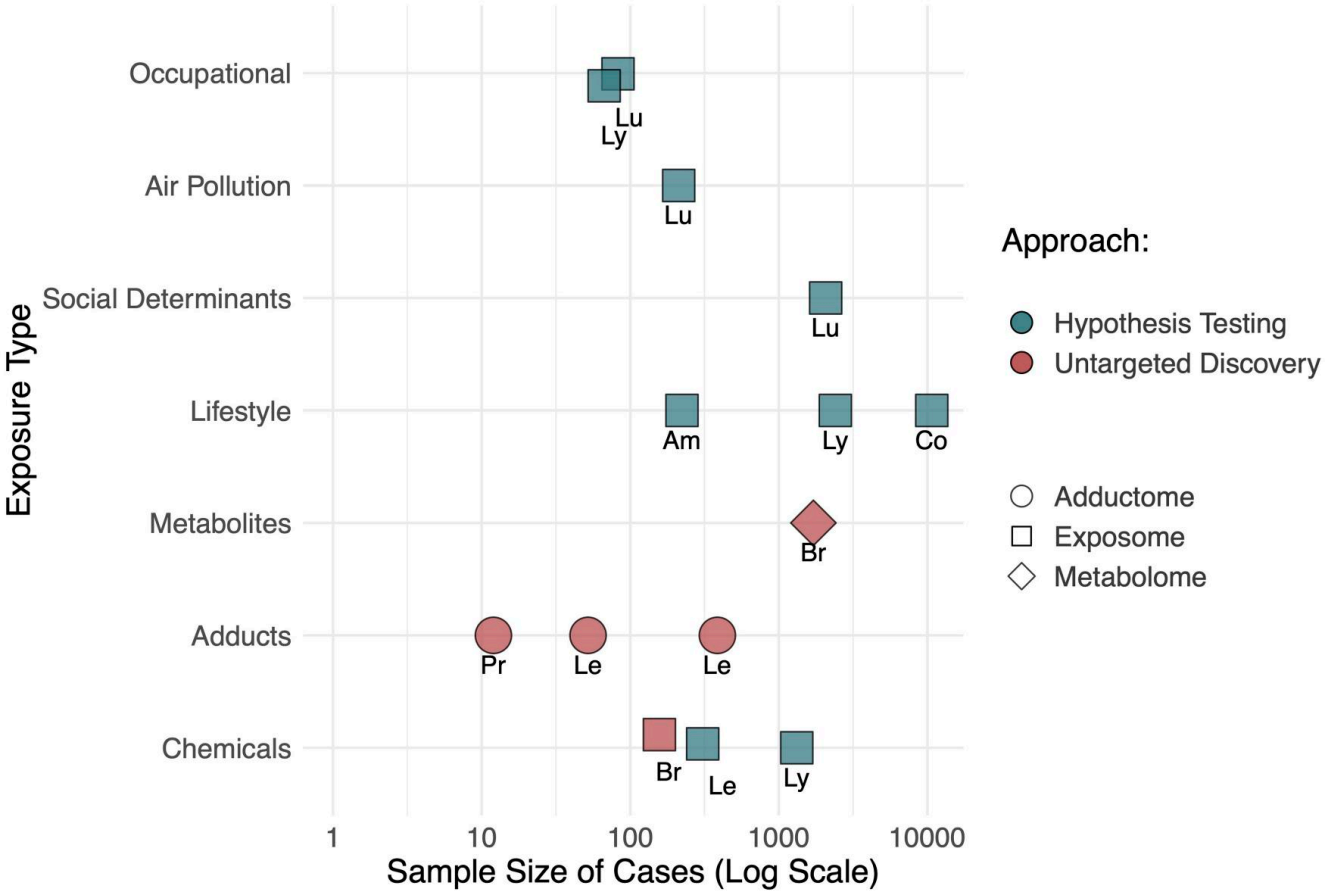
Summary of study designs and time period that exposure data reflects relative to cancer outcome occurrence for the 14 eligible studies included in our scoping review. Note: One study with cross-sectional exposure data at time of outcome also asked about pre-outcome factors.

Most of the study designs were nested case–control, case–cohort, or cohort samples with prospective exposures before diagnosis (43%: 6 studies) (Table 1; Figure 4). Two ecologic study designs (evaluating county-level or ZIP code–level data) had prospective exposure variables (13%). The other study designs were either case–control or case-only (43%: 6); among these, only 2 studies had exposures reflecting time before the outcome (whether assessed prospectively or retrospectively). Among all study designs, 10 studies (71%) used exposures before the outcome, 3 studies (21%) used exposures reflecting a time period after the outcome, and 1 study (7%) used exposure information at the time of outcome occurrence. Only 1 study analyzed longitudinal exposure data, although the repeated serum samples across 2 trimesters of pregnancy were analyzed independently. Total sample sizes for final exposome-related analyses ranged from 12 to 475 426 (median: 726), including cases and controls. Among the 12 nonecologic studies, cancer case counts ranged from 12 to 10 702 (median = 246). Four nonecologic studies (29%) had case counts less than 100, and another 4 studies (29%) had less than 400. Among only the 4 nonecologic studies described as “exposome” or “adductome” and that analyzed biospecimens, the case counts were all below 400 (Table 1).

#### Exposure assessment

Among the 14 eligible exposome-relevant studies, 10 studies (71%) purposely focused on the exposome, 3 studies (21%) measured the adductome, and 1 study (7%) used metabolomic methods (with intentional assessment of environmental exposure). Although the 5 hypothesis-discovery studies sought to quantify known and uncharacterized environmental chemicals, adducts, or their metabolites in biospecimens, the 9 hypothesis-driven exposome studies assessed exposures related to primarily lifestyle factors (3 studies), known targeted chemicals in house dust (2 studies), occupational factors (2 studies), air pollution (1 study), and social determinants of health (SDOH) (1 study) (Figure 3; Table 1). Nonlaboratory sources of exposure variables included self-reported questionnaires, medical records, job information, geospatial data, and chemical emissions data.

Of the 10 studies with methods specifically described as exposomic (not adductomic or metabolomic), 9 studies (64%) used mainly hypothesis-driven approaches (selecting exposure hypotheses a priori), and only 1 study used purely hypothesis-discovery (untargeted) methods (Figure 3; Table 1). This untargeted chemical exposome study employed LC-HRMS as the analytical platform. None of the studies used untargeted GC-HRMS. Among the 9 hypothesis-driven exposome studies, a median of 49 exposure variables were analyzed (range = 3–2162), with 8 studies examining fewer than 110 exposure variables (Table 1). The study with 2162 external exposure variables had a hybrid objective of both hypothesis testing and hypothesis generation. None of the studies conducted multi-omic integration, although in 2 studies, untargeted LC-HRMS analysis naturally captured overlap between the exposome and metabolome.

## Discussion

### Summary of findings

Exposome research presents a unique opportunity to identify environmental risk factors that can be targeted for intervention in the prevention and control of cancer, for which an estimated 90% of incidence is driven by nongenetic risk factors.^4^ In our scoping review of 14 studies published before August 2023, we found that research on the human cancer exposome is growing but still limited. Importantly, only a handful of studies employed an untargeted, hypothesis discovery approach using human biospecimens. The 1 cancer study that specifically measured the untargeted chemical exposome (using LC-HRMS) demonstrated the power of the technology to identify previously uncharacterized exposure risk factors and their potential mechanistic pathways within an epidemiological framework.^37^ However, none of the studies have used GC-HRMS or multiple analytical platforms for the untargeted exposome. As a different approach, 3 cancer studies leveraged adductomics to measure cancer-related transformation products of environmental chemicals that bind to blood proteins or DNA. Most other eligible studies in our scoping review employed a hypothesis-driven approach with external exposure variables selected a priori for testing. These external exposures covered a range of factors at varying scales, including targeted chemicals, self-reported lifestyle, occupational history, geospatial measurements, and, in 1 case, county data on SDOH. The study data sources highlight the variety of interdisciplinary methodologies available to capture external exposures to environmental factors across different micro-environments and systems.^47^ Study designs were mixed, but a considerable number of studies could not use *pre*-diagnosis information on exposures and/or access more than a few hundred cancer cases, and there was a scarcity of longitudinal exposure data collection. The application of the exposome to different types of cancer is growing, but only 6 of more than 200 types of cancer have been covered so far,^4^ and only in high-income countries. Overall, our scoping review highlights the critical need to advance the study of the cancer exposome and identifies a scarcity of prospective or longitudinal cohorts utilized to interrogate the cancer exposome.

**Figure 5.**
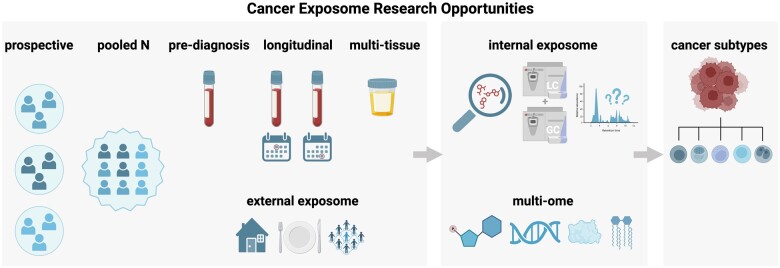
Research needs and study design considerations for the cancer exposome. Note: icons are for illustration purposes and not intended to be comprehensive. Here, the external exposome refers to environmental stressors occurring outside the body, while the internal exposome refers to biomarkers of internalized exposures to those environmental stressors.

To further advance exposomics as a key tool for cancer prevention and control, in the next section we focus on unmet opportunities and challenges in cancer exposome research (Figure 5): (1) underuse of the internal chemical exposome integrated with the external exposome and other -omes to systematically characterize exposures associated with cancer outcomes; (2) study design considerations; (3) current challenges in exposome methodology, including chemical coverage, chemical annotation, chemical concentrations, and cross-study harmonization; and (4) potential for a “cancer exposome atlas” that pools resources across populations and across cancer types.

### Underutilized exposome approaches

#### Internal chemical exposome

Only 1 study in our scoping review investigated the untargeted internal chemical exposome, specifically for breast cancer risk. Untargeted profiling of chemicals via exposomic methods is a powerful tool to assess exposures to complex mixtures of *tens of thousands* of environmental chemicals.^23^ Untargeted methods do not require a priori selection of chemical analytes and thus can yield new hypotheses and discoveries beyond what we can anticipate. More than measuring the original chemicals, the internal exposome reveals their biotransformation products, which reflect an individual’s unique physiology and are often responsible for inducing the toxic effects.^13^^,^^48^ This avenue of research also provides an objective, quantitative measure of exposure not based on traditional self-report or subjective assessments with potential for recall bias.^49^ Several important methodological choices can optimize the detection of the highly diverse space of environmental chemicals, such as the use of multiple instrument platforms^20^—see “Current challenges in untargeted exposome analytics”.

#### Functional exposomics

To improve assessment of *root causes* of internalized exposure, Zhang et al. proposed a “functional exposomics” approach that bridges external exposure measures of external environmental stressors with internal dose and biological response.^13^ Traditionally, studies—including most in our review—have taken either a top-down approach (internal exposome, for better links to health) or bottom-up approach (external exposome, for better links to sources).^14^^,^^50^ An ideal functional exposomics study would include measures of both the internalized biomarkers of or response to exposure and the external sources of exposure in an individual’s environment.^13^ On one hand, the internal exposome excels at capturing internalized manifestations of environmental exposure (including nonchemical stressors) in an integrated manner. However, internal biomarkers of the exposome may not always represent the original parent chemicals, capture exposures that compartmentalize into different tissues for different durations, or discriminate between exposures with shared biotransformation pathways, which can limit interpretation for interventions and require more experimental knowledge on biological response.^51^ On the other hand, the external exposome provides insight into direct sources and classification of exposures even if they are not associated with specific or long-lasting enough biomarkers; however, external exposure measurements do not capture the dose that actually reaches target tissues in the body. External exposure is extremely expansive in scope: measurement strategies include questionnaires, environmental media (eg, air, soil, dust, products), geospatial methods, remote sensing, smartwatches, or personal external sampling devices, such as silicone wristbands.^13^^,^^24^^,^^29^^,^^52-54^ In summary, an array of external and internal exposure measurement techniques exist to support the aim of the exposome to “integrate data from a variety of interdisciplinary methodologies and streams.”^47^

#### Social determinants of health

SDOH are a defining but often overlooked driver of external exposure and resulting health inequities. The external exposome originates from a complete taxonomy of natural, built, social, and policy environments, including systemic-level stressors (eg, racism, discrimination, neighborhood deprivation, and healthcare access).^55-57^ SDOH can physicalize in the body and add to cumulative health burden (“allostatic load”).^58^^,^^59^ In one way, SDOH powerfully predict disproportionate exposures to certain harmful chemicals, such as from beauty products.^60^^,^^61^ In another, social stressors may induce intracellular damage, oxidative stress, chronic inflammation, immune aging, and low immune capacity, increasing susceptibility to cancer and disease.^56^ Other biological responses to SDOH include alterations to gene expression and methylation, sex hormone signaling, cortisol responsiveness, adrenaline levels, heart rate, blood pressure, brain function or plasticity, and neurotransmitter concentrations.^58^

Exposome approaches hold the opportunity to measure SDOH (and their biological responses) at a wider scale and determine mediators between SDOH and cancer. In our review, only 1 cancer exposome study focused on social determinants of disparities,^40^ and racially/ethnically diverse cohorts are critically needed to support investigations of cancer disparities.^62^ Systemically marginalized populations face pronounced disparities across the continuum of cancer from screening to diagnosis to survival. For prostate cancer, Black men experience worse incidence rates, clinical presentations, treatment quality, and mortality rates than White men in the United States.^63^ Cancer is the leading cause of death among Asian Americans, unlike other racial and ethnic groups in the United States, yet they have lower rates of cancer screening and likely receive fewer provider recommendations for screening.^64^ Although breast cancer incidence rates are similar between Black and White women in the United States, Black women experience more delayed treatment, more failure to receive appropriate treatment, and lower survival rates at every age.^65^ Global disparities also exist for common cancers, with lower incidence rates but poorer survival rates among low- and middle-income countries compared with high-income countries.^66^ In our review, studies evaluated cohorts only from the United Kingdom, the United States, and high-income countries in Europe. 

In summary, the exposome offers a powerful approach to unravel complex interactions between *multiple* co-occurring determinants of cancer disparities.^39^ Functional exposomics could quantify internal biomarkers of both chemical exposure and biological responses to social determinants and investigate their interaction in cancer initiation. SDOH research will require intentional study designs enhancing inclusion of systemically marginalized or underrecognized populations, with the ultimate goal of informing *equitable* prevention strategies for cancer.

#### Multi-omic integration

Cancer initiation is a complex process occurring across multiple biological layers.^4^^,^^14^ As such, we need cancer research that investigates the complex interactions of the exposome across biological layers within a so-called multi-omic framework. For example, key characteristics of carcinogens include genotoxicity (eg, the genome), altered gene expression (the epigenome and transcriptome), metabolic activation (the metabolome), cell proliferation with altered energetics (the lipidome), and disruption of receptor proteins and signaling (the proteome) among other properties, each of which have interactions, crosstalk, and feedback across multiple “-omes.”^4^^,^^16^^,^^67^ Yet our scoping review revealed no cancer study so far that has integrated exposome profiling with the genome, epigenome, transcriptome, proteome, or lipidome.

A multi-omic framework can elucidate not only traditional genotoxic mechanisms but also nongenotoxic mechanisms of environmental carcinogens. Predicting nongenotoxic carcinogenicity is difficult given their varied modes of action and specificity to tissues and species.^15^ For instance, polychlorinated biphenyls are a group of chemicals that tested negative in most genotoxicity assays and operate through numerous other tumor-promoting mechanisms, but individual polychlorinated biphenyl congeners can differ in their dependence on the aryl hydrocarbon receptor for carcinogenicity.^68^ What’s more, even noncarcinogenic chemicals may act with low-dose effects on pathways related to carcinogenesis such that their cumulative effects with other noncarcinogenic chemicals could plausibly contribute to carcinogenesis.^69^^,^^70^ The complexity of chemical carcinogenicity urges multi-omic cancer research that integrates the untargeted exposome with an array of biological response networks. This approach would aid in identifying environmental carcinogens, mechanisms of action, therapeutic targets, and biomarkers of disease or prognosis.

The complexity and high dimensionality of exposome and multi-omic data justify specialized statistical analysis strategies to evaluate associations with disease or mechanisms, given the extreme number of untargeted exposure variables and their high correlations (eg, shared sources) and interactions with one another. However, there exists little standardization in statistical approaches for the exposome or its multi-omic integration, with dramatically higher numbers of parameters.^71^^,^^72^

“Meet-in-the-middle” (MITM) strategies can identify associations between the exposome and disease outcomes and determine which biological response pathways fall along those associations, although studies approach MITM differently.^71^ For example, in integrating the exposome and metabolome, a basic concept is to (1) identify exposures significantly associated with disease, (2) determine pathways of metabolites significantly associated with disease, and (3) network the intersection between disease-associated exposures and metabolic pathways, thus identifying which metabolic mechanisms intersect between exposure and outcome.^71^ The first step is a typical exposome-wide association study that screens untargeted chemicals nominally associated with disease on the basis of many separate single-exposure regression models.^73^ Regardless of statistical method, multiple testing of potentially thousands of biomarkers must be corrected post hoc to balance false positives with false negatives (eg, by Bonferroni or less conservative Benjamini–Hochberg approaches).^71^ Study design must also be considered, including potential reverse causality in cross-sectional multi-omic studies. MITM methods would be best interpreted with prospective prediagnosis sampling and longitudinal measures of the intermediate biomarkers. Other approaches that integrate multi-omic (or “multiview”) data can identify biological mediators of risk or improve predictive accuracy of disease risk but make varied assumptions about the relationships and differences between multi-omic layers.^74-78^ Those methods usually conduct “early” integration of multi-omic data, such as by first concatenating the layers into 1 matrix before inference; “late” integration, which performs inference on the separate layers independently before aggregating features; or “intermediate” integration, which leverages a joint model to combine layers.^75^

Numerous mixture methods have been developed to model combined health effects from simultaneous exposures to multiple environmental factors (the “mixture”), although not all approaches scale to -omics levels. Mixture methods include weighted quantile sum regression with repeated holdouts (WQS_RH_),^79-82^ quantile-based g-computation (qGc),^83^ and Bayesian kernel machine regression (BKMR),^84^^,^^85^ among many others.^86-89^ In the -omics case when the number of chemical predictors greatly exceeds the number of participants, the “random subsets” variation of WQS implements feature bagging wherein a smaller subset of chemicals is randomly selected for estimation and repeated many (eg, 1000) times to de-correlate the data, avoid co-confounding, reduce overfitting, and improve generalizability.^80^ In general, choice of mixture method depends on the designed research question, whether it be overall effect estimation, single effect estimation, pattern identification, toxic agent identification, or assessment of interactions and nonlinearities.^86^ Other approaches seek to reduce the data dimensions by condensing exposures into a more manageable number of factors, although often with sacrifices to interpretability.^86^^,^^90^ Network-based approaches have been proposed as more biologically relevant than purely statistically determined data reduction; for example, correlation or partial-correlation networks can identify functional relationships between features and also group features into clusters for further analysis.^91^ Finally, aside from exposome-wide analyses, approaches for outcome-wide exposome analyses offer capabilities to investigate exposures that simultaneously impact multiple diseases or exposures that are protective for some outcomes but risk factors for others.^92^

#### Exposome risk scores

Similar to polygenic risk scores that estimate risk on the basis of a combination of genetic mutations, exposome risk scores aim to summarize how a mixture of environmental, nongenetic factors combine to increase disease risk.^21^^,^^88^ Such a precision exposome approach could help inform an individual’s prevention or treatment strategies, identify susceptible groups at high risk, reveal hotspots of concern from combinations of environmental risk factors, and prioritize risk factors for intervention.^21^^,^^93^^,^^94^ Unlike polygenic scores, exposome risk scores may also reveal modifiable sources for disease prevention, although not all exposures are necessarily modifiable or causal.^93^ To date, risk scores have yet to be applied to cancer exposome research.

The internal exposome’s molecular snapshots of an individual’s recent exposure history based on biomarkers in biospecimens^95^ could also offer information not available from questionnaires, especially for biorepositories unable to re-survey participants. A proof-of-principle study identified internal metabolomic biomarkers of tobacco smoke exposure on the basis of experiments testing blood of smokers before and after each cigarette.^96^ However, not all external exposure sources may be directly internalized or characterizable as metabolites, let alone as unique metabolites.^97^

### Study design considerations

#### Large-scale, prediagnosis, and longitudinal sampling

*Large sample sizes*. In exposome or metabolome studies, sample size tends to be orders of magnitude lower than the number of detected chemicals or metabolites. In a recent review, most cancer metabolomics studies had fewer than 300 cancer cases, and only 5 studies had more than 1000 cases (the highest had approximately 3000).^62^ Given that more than 100 000 untargeted signals are detectable in human blood and more than 50 000 can be annotated (based on our laboratory’s recent analyses of nested case–control cohorts), large sample sizes are essential to accommodate data science approaches and improve statistical power to detect associations. For example, a study found considerable temporal variability in plasma levels of as few as 385 metabolites within an individual and estimated that, after accounting for several sources of variability, epidemiologic case–control studies (with 1:1 matching) would need large sample sizes to detect disease risk. Specifically, with 5000 samples, an estimated 97% of studies could detect a 2-fold relative risk of disease (comparing top and bottom quartiles of a metabolite), whereas only 38% of studies with 500 samples could detect an association of that magnitude after correcting for multiple testing.^98^ As a result, environmental chemicals with small effects on cancer risk may go unrecognized. Larger sample sizes would also enable separate analysis of molecularly defined *subtypes* of cancer instead of only considering the primary cancer type.^99^ With specimen biobanks and with untargeted exposome technologies becoming more automated, high-throughput, and affordable,^14^ studies will be able to analyze higher sample sizes.

*Prediagnosis sampling.* Prospective prediagnosis samples for exposure characterization are critical for identifying *causal* risk factors, compared to traditional cross-sectional or case–control sampling done at the same time as the outcome. For one, cancer has a long latency period with multiple steps in its progression, and the time between cancer initiation and diagnosis sometimes takes decades.^100^^,^^101^ As a result, the disconnect between early exposure versus exposure at time of diagnosis can lead to misrepresentation of the actual carcinogenic exposure and generate reverse causation, which occurs when preclinical symptoms, treatment, or subclinical disease processes precede and influence observed exposure.^102^ This limits inferences of causality. Furthermore, cross-sectional designs may oversample cases alive with cancer for longer durations (ie, less rapidly fatal cases); then exposures that influence only *mortality* of cancer in either a positive or negative direction may show spurious associations with diagnosis of cancer.^102^ By contrast, study designs using baseline samples from a prospective cohort or its nested case–control/case–cohort subset capture more representative exposure windows before disease. The use and development of large biobanks are key for ensuring resources to research cancers with long latency periods, and continuation of data collection for preexisting biobanks and repositories represents an important priority in exposome epidemiology.^6^ In research of the *mechanisms* of disease, cross-sectional associations between the exposome and metabolome simultaneously measured in a single sample have offered a convenient systems-biology approach^20^^,^^29^; however, special care must be taken because of the potential for misclassified exposure and reverse causation here as well. Ideally, repeat samples would be collected to investigate how the exposome influences *changes* in the metabolome or other -omes.

*Longitudinal sampling.* Unlike the genome, the exposome is highly dynamic, unfixed in time, and sometimes short-lived in the body.^23^ Because of uncertainty about the reliability of -omic measures over time,^103^ repeat longitudinal sampling would build a fuller picture of someone’s exposure history during critical life stages.^32^^,^^50^ Longitudinal analysis could also improve detection of associations between exposure and cancer, as carcinogens may affect cancer at different stages during its long latency, including the initiation, promotion, or progression phases.^15^ Repeat samples would also enable investigation of intermediate biological response pathways between the exposome and outcome without relying on cross-sectional data. Finally, analyzing multiple samples per individual notably increases statistical power in -omics studies by reducing within-individual variability and technical variability in metabolites.^98^ However, large-scale prospective longitudinal studies are expensive and labor-intensive, so other study design approaches are important sources of information on potential environmental risk factors for further mechanistic investigation. Very little published research has assessed longitudinal exposome changes and disease risk,^104^ and no cancer exposome studies in our review have done so.

### Current challenges in untargeted exposome analytics

#### Chemical coverage

To enhance the coverage of highly diverse environmental chemicals and their endogenous metabolites, multiple instrument platforms are needed, such as both LC-HRMS and GC-HRMS.^19^^,^^20^ These complementary strategies account for the fact that traditionally measured chemicals occur at concentrations typically 100-1000 times lower than endogenous metabolites and span about 18 orders of magnitude in water solubility, with around half more amenable to GC than LC analysis, whereas their biotransformation products are best detected by LC.^13^^,^^23^ However, most studies employed a single instrument platform, including the 1 untargeted chemical exposome study (using LC-HRMS) in our scoping review.^24^^,^^105^ Recent and ongoing improvements in high-throughput automation and lower sample volumes with HRMS will help support multi-instrument analysis at less expense with faster workflows.^14^ To further augment the coverage of chemicals that vary in polarity, lipophilicity, and half-lives, studies can consider analyzing multiple tissue compartments, such as blood, urine, and tumor tissue and multiple assay parameters.^14^^,^^24^^,^^106^ Ultimately, no approach is completely comprehensive, and the final choice requires trade-offs regarding the properties of chemicals for which detection is optimized.^24^

#### Chemical annotation

A major bottleneck in exposome analysis is identification of detected mass spectral signals.^6^ Although HRMS methods have the power to detect upwards of 1 million signals in human blood,^12^ the vast majority of detected chemical signatures remain unannotated as the “dark matter” of the exposome or metabolome.^107^ Laboratory output provides a mass-to-charge ratio (*m/z*), ion fragmentation (MSMS) spectra (for a limited number of peaks), and retention time of each chemical feature, which are cross-matched against in-house mass spectral libraries from standards (which provide confirmed identifications) or external libraries and databases for annotations (which assign confidence levels to possible identities based on MS information such as mass defect, retention time, intensity profiles, isotope/adduct patterns, and metabolic pathway associations).^14^^,^^108^ For example, the Blood-Exposome Database provides 2-dimensional structures for around 40 000 chemicals seen in blood.^109^ The confidence levels associated with annotation of a particular detected feature can be highly variable between one feature and another, and from one study to another, making interpretations difficult.^110^ Pathway enrichment analysis is a supplemental approach to predict biological activity from networks of detected metabolites without needing to identify everything.^111^^,^^112^

Importantly, even when a chemical feature cannot be confidently identified, it can still reveal structural information and be statistically analyzed for associations with disease. Advancement and standardization of cheminformatic algorithms will be important not only for chemical annotation but also across the full data pipeline, including peak picking, peak alignment, and pathway analysis, to support generation of harmonized data.^20^^,^^28^

#### Study harmonization

Untargeted exposome assays report detected signals as ion intensities,^23^ unlike targeted chemical analysis wherein isotopically labeled internal standards are combined with external calibration curves defining the relationship between peak intensity and compound concentration. However, targeted analysis would not be possible or financially feasible for thousands of characterized and uncharacterized chemicals.^113^ Although ion intensities of untargeted chemicals are internally valid as relative abundances that can be compared between participants within the study, they cannot be compared to exposure levels in other studies or to health benchmarks.

Reference standardization is a highly feasible approach that supports calculation of chemical concentrations in high-throughput, untargeted workflows. Pooled reference samples that were previously fully characterized are analyzed before and after each sample batch and provide a basis against which to reference sample ion intensities and a strategy for calculating analyte concentrations using single-point calibration.^23^^,^^26^^,^^114^ In this way, only the pooled reference samples need targeted concentrations, and the analytes for participant samples still do not need to be chosen a priori.^23^ In some cases, chemical quantification could even be conducted retrospectively as more features are characterized in the study samples and identified in the stable pooled references.^115^ For chemicals without standards, reference samples can also help harmonize data across laboratories by providing a common material to normalize ion intensities detected in samples.

Harmonization of studies to support exposome data integration across cohorts is challenging given these uncertainties in chemical annotation, measurement of ion intensities, and heterogeneity in analytical approaches. Harmonization for better comparability and interpretation of results will require establishing criteria for standardization, consistency, and acceptability across the entire analytical workflow (including sample collection, sample preparation, instrumental analysis, data processing, feature detection, and annotation).^116^ Recent attempts to harmonize human exposome data across several laboratories have demonstrated promise.^117^

### Need for a “cancer exposome atlas”

To equip studies with the sample sizes to investigate the full complexity between the exposome (with tens of thousands of biomarkers) and cancer (with over 200 types in addition to subtypes), a global cancer exposome atlas at the same scale as the Cancer Genome Atlas Program (TCGA) is urgently needed. TCGA was jointly formed in 2006 by the National Cancer Institute and National Human Genome Research Institute and molecularly characterized paired tumor and normal tissue samples from more than 11 000 patients across 33 different types of cancer.^1^^,^^118^ Large-scale repositories are being established and will offer avenues for pursuit of an exposome atlas. The International Hundred Thousand Plus Cohort Consortium has brought together more than 100 cohorts across 43 countries that each target the recruitment and biological sampling of at least 100 000 participants, resulting in a registry with nearly 50 million participants.^119^ Such large sample sizes that better match the number of predictor variables will accomplish the statistical power to uncover small effects of countless untargeted environmental exposures on cancer, the case counts to evaluate rare subtypes of cancer, and the population diversity to assess SDOH and social determinants of exposure. The cancer exposome will require expanding beyond researching only 1 exposure at a time or 1 cancer at a time and toward pooling studies into a cumulative resource across populations and across diseases. Although the pooling of heterogenous cohort data comes with limitations, harmonized laboratory methods for biospecimen analysis will enhance the comparison of exposome measurements in pooled cohorts. Such an exposome atlas enables a streamlined, agnostic approach for the discovery of novel environmental risk factors and biological pathways in disease, which can then inform targeted research diving deeper on specific hypotheses.

### Limitations of review

Our scoping review was limited to published papers appearing in 3 leading databases. Our strategy did not capture gray literature, unpublished work, or non-English articles. Finally, we did not evaluate the findings, statistical approaches, study design quality, or potential biases in this review.

## Conclusions

This scoping review summarized exposome definitions, technological advances, methodological challenges, and study designs, which are relevant for the study of any disease. We examined critical gaps in literature applying the exposome to cancer specifically. Discovery-based approaches, particularly the untargeted chemical exposome, present a largely unmet opportunity to identify previously uncharacterized environmental risk factors and inform primary prevention of cancer. The chemical exposome better represents the full complexity of our environmental exposure to characterized and uncharacterized chemicals, although the high-dimensional data require specialized study design considerations and statistical approaches. Continued technological development for untargeted exposomics will seek to overcome limitations related to optimized chemical coverage, chemical identification, concentration estimation, and cross-study data harmonization. Other research gaps include the functional (hybrid internal/external) exposome, multi-omic integration, and social determinants of the cancer exposome. Future studies should strive for larger sample sizes, more diverse cohorts, cancer subtype analyses, prediagnosis exposure sampling, and longitudinal sampling. Efforts toward a global “cancer exposome atlas” at a scale akin to the genome atlas would support hypothesis generation across many populations and cancer types with high statistical power. In summary, the exposome offers a powerful tool to study environmental risk factors at the true level of complexity underpinning cancer development.

## Supplementary Material

pkae122_Supplementary_Data

## Data Availability

The data underlying this article are available in the article and in its online supplementary material.
