# Supplementary material for: The need for a cancer exposome atlas: a scoping review
Source: JNCI Cancer Spectr. 2024 Dec 19;9(1):pkae122. doi: 10.1093/jncics/pkae122 (PMC11729703; doi:10.1093/jncics/pkae122)
Supplement: pkae122_Supplementary_Data [file pkae122_supplementary_data.docx]

**The Need for a Cancer Exposome Atlas: A Scoping Review**

*Supplementary Material*

Scoping Review Methods

***Eligibility Criteria***

We included published (or pre-print) scientific journal manuscripts of original research evaluating the exposome in relation to outcomes of cancer in human participants. For exposome eligibility, we focused on studies specifically described as the “exposome” (whether chemical or non-chemical), and we further included studies measuring untargeted chemicals, semi-untargeted chemicals (suspect screening), or chemical adducts without using the term “exposome”. Thus, in the context of this review, we considered exposome studies to be those assessing *environmental exposure* using any of the following methods: (1) specifically described as exposomic in the title or abstract (regardless of the definition or use of biological specimens), (2) untargeted chemical analysis, (3) suspect screening chemical analysis, (4) untargeted chemical adductomics, or (5) untargeted metabolomics with discussion of identified chemicals in abstract, where we define “chemicals” as exogenous xenobiotic chemicals. This approach thus included studies that did not refer to (or did not know to refer to) their exposomic methods as such. Adductomics refers to the measurement of adducts formed when reactive electrophiles generated from the metabolism of chemicals bind to blood proteins or DNA (19,20). The microbiome was outside the scope of our exposome review. We only included papers written in English. We excluded sources that were: not original research presenting data (e.g., review, meta-analysis, cohort methodology, perspective, commentary, or editorial); not full journal papers (e.g., conference papers or abstracts); did not involve human data (e.g., *in vitro* or animal studies); or did not evaluate cancer as an outcome in human participants (e.g., study of only exposure or of other diseases; study of cancer patient population but not evaluating associations with cancer-related outcomes). During the full-text review, we screened out metabolomics studies that did not address or intend to assess environmental exposure with their methods, even if in the results or discussion sections the authors identified environmental chemicals that they happened to detect in samples.

***Search Strategy***

We identified studies in the following bibliographic databases: PubMed/MEDLINE, Embase, and Web of Science: Core Collection. The search query was drafted and refined in discussion by a team of four study authors (AY, CM, NS, and DW). The results of the database searches were batch exported into RIS or PubMed formats and then imported into the review management software Covidence (operated by Veritas Health Innovation Ltd). Covidence automatically removed duplicate sources.

The database search was performed by AY on 10 August, 2023. The searches were filtered to English language, with no lower limit on publication year. The search query was applied to only the title (ti), abstract (ab), and author keywords (kw) of articles. The generalized search query was as follows: *(( exposom* OR adductom* OR metabolom* OR untargeted OR nontargeted OR “non targeted” OR “non-targeted” OR “suspect screening” ) AND ( expos* OR environment* OR chemical* OR xenobiotic* OR genotoxin* ) AND ( cancer* OR tumor* OR tumour* OR malignanc* OR neoplasm* OR lymphoma* OR myeloma* OR leukemia* OR leukaemia* ) AND ( exposom* OR adductom* OR sample* OR biospecimen* OR specimen* OR blood OR plasma OR serum OR urin* )):ti,ab,kw NOT ((“in vitro” OR mice OR murine OR mouse OR rat OR rats):ti)*. The second clause of the query refined results to those mentioning cancer or other cancer outcome terminology to maximize literature coverage. The last two clauses in the query were intended to help refine results to human sampling (i.e., not *in vitro* human cell lines or animal experiments) for any articles that did not already specifically refer to the exposome or adductome. For Web of Science, we changed the search query fields in our registered protocol so as to not search within their KeyWords Plus® (which are generated based on common terms used in titles of the references *cited by* the article and not based on the actual content of the article), and rather only use author-provided keywords as the other databases do.

***Study Selection***

In Covidence, each study’s title and abstract were screened by two reviewers. Any disagreements in study inclusion were independently resolved by a third reviewer, with discussion and consensus as needed. The same process was then conducted for the full-text screening phase. The reviewers were AY, CM, and NS.

Before screening, we performed a calibration pilot test by all three reviewers using a random sample of 50 citations. The team screened the 50 titles/abstracts and extracted data for an eligible full-text study. Discrepancies were discussed between the reviewers, and the screening criteria checklist and data charting form were refined as needed. For example, in the inclusion/exclusion criteria, we clarified that we were not considering microbiome exposures in this review, that the requirement for human sampling included data collection from surveys and other non-biospecimen sampling of humans, and that the exposome requirement includes any study that specifically reports to measure the “exposome” regardless of their actual approach to do so. We also refined the search query to include its final clause that helps further exclude studies only focused on *in vitro* or animal experiments, as there were many such studies in the pilot screening.

***Data Extraction and Synthesis***

Data extraction of the screened full-text articles was conducted in the calibrated data charting form that we designed within Covidence. Two reviewers independently performed data charting. Any discrepancies were resolved by discussion between the reviewers and resolution by a third reviewer as needed. The questions in the form were posed with pre-filled answer choices when possible, as well as “other” options. Our data extraction focused on the parts of the study methods that were relevant to the topic of our scoping review.

For results, we grouped the included studies by the exposome approach, exposure types, exposure timing, cancer outcome measure, and epidemiologic study design. We summarized the characteristics of the studies in descriptive formats, tables, and diagrams.
